# Discharge Against Medical Advice Among Schizophrenia Patients in Germany: A Multicenter Cross-Sectional Study

**DOI:** 10.3390/brainsci15020196

**Published:** 2025-02-14

**Authors:** Karel Kostev, Ira Rodemer, André Hajek, Marcel Konrad, Lee Smith

**Affiliations:** 1 Epidemiology, IQVIA, 60549 Frankfurt, Germany; 2 University Clinic, Philipps-University, 35043 Marburg, Germany; 3 Department of Health Economics and Health Services Research, University Medical Center Hamburg-Eppendorf, Hamburg Center for Health Economics, 20251 Hamburg, Germany; 4 Department of Health and Social Studies, FOM University of Applied Sciences for Economics and Management, 60486 Frankfurt, Germany; 5 Centre for Health, Performance and Wellbeing, Anglia Ruskin University, Cambridge CB1 1PT, UK

**Keywords:** schizophrenia, discharge against medical advice, psychiatry, hospital

## Abstract

Background: The objective of this study was to investigate the prevalence of discharge against medical advice (DAMA) among schizophrenia patients in Germany and to identify factors associated with the risk of DAMA. Methods: This multicenter cross-sectional study was based on data from the IQVIA hospital database, which contains records from 36 hospitals across Germany. This study included all hospitalizations for patients with a primary or secondary diagnosis of schizophrenia between January 2019 and December 2023. Multivariable logistic regression analyses adjusted for age, sex, primary or secondary schizophrenia diagnosis, as well as codiagnoses, were conducted to assess the associations between demographic and clinical variables and DAMA. Results: A total of 7663 hospitalization cases (mean age: 49.5 years, 40.6% female) were included in the study. The DAMA rate was 31.1% in patients with schizophrenia as the primary diagnoses and 6.0% in patients with schizophrenia as a secondary diagnosis. Younger age (i.e., adjusted odds ratio (aOR): 7.44; 95% CI: 4.35–12.73 in the age group 18–30; aOR: 6.63; 95% CI: 3.89–11.29 in the age group 31–40; aOR: 5.59; 95% CI: 3.28–9.54 in the age group 41–50), schizophrenia as the primary diagnosis (aOR: 3.61; 95% CI: 3.05–4.26), alcohol-related disorders (aOR: 1.68; 95% CI: 1.38–2.04), and cannabis-related disorders (aOR: 1.43; 95% CI: 1.18–1.72) were significantly associated with an increased risk of DAMA. Conclusions: This study highlights the high prevalence of DAMA among hospitalized schizophrenia patients and identifies the important factors (i.e., younger age, alcohol-related disorders, and cannabis-related disorders) associated with DAMA risk. Additional studies are recommended for further exploration into the reasons for DAMA.

## 1. Introduction

Discharge against medical advice (DAMA) occurs when patients opt to leave the hospital contrary to the recommendation of their treating physician [1]. A cross-sectional analysis of inpatient hospitalizations between 2011 and 2022 in the United States revealed a DAMA prevalence of 1.1%, with higher rates in men, young patients, and patients with minor illness severity [2]. DAMA poses unique concerns among psychiatric patients due to the nature of their illness, which often includes impaired judgment and cognitive deficits.

Schizophrenia is a severe mental disorder characterized by disruptions in thought, perception, and behavior. It typically requires long-term treatment, often involving antipsychotic medication, psychotherapy, and social support to manage symptoms and prevent relapses [3]. Individuals with schizophrenia may be at a high risk of DAMA, often due to the lack of insight into their illness [4,5].

There is lack of studies investigating DAMA in schizophrenia patients, and the findings were inconsistent. In a database study conducted in the US, which included 210,722 schizophrenia patients, 1.6% of admitted patients were discharged against medical advice [6]. In another study conducted in Israel, based on 12,937 hospitalizations of schizophrenia patients, 8.3% were discharged against medical advice [5]. In a single-center study from a major psychiatric center in Taiwan, which included 637 schizophrenia patients, the DAMA rate was 18.7% in the total study population and 23.2% in male patients [7].

In previous studies, schizophrenia patients discharged against medical advice were significantly younger and more likely to be male [5,6]. Patients with schizophrenia who are discharged against medical advice have higher readmission rates and elevated mortality rates, often due to suicide and accidents [7,8].

Understanding the prevalence and predictors of DAMA among schizophrenia patients in Germany is crucial for developing strategies to address this issue and enhance patient outcomes. The majority of studies on DAMA have examined prevalence prior to 2010 [4,5], and their findings may not accurately portray recent changes. Furthermore, a substantial proportion of studies were conducted in the US [6] and Asia [7,8] and it may not be possible to extrapolate their findings to Germany, owing to differences in hospital policy and healthcare system structures, for example.

Therefore, the objective of this study was to assess the prevalence of DAMA among schizophrenia patients in Germany and to identify the demographic and clinical factors associated with the risk of DAMA.

## 2. Materials and Methods

### 2.1. Data Source

This multicenter cross-sectional study utilized data from the IQVIA hospital database, which contained data from 36 hospitals across Germany, including specialized hospitals, primary care hospitals, maximum care, standard care, and university hospitals. Data were available for a total of 1,223,696 hospitalizations in the period from 2019 to 2023. The data set, known as Section 21, includes information in a standardized data format, transmitted by hospitals to the Institute for the Hospital Remuneration System (InEK) in accordance with Section 21 of the German Hospital Fees Act (Krankenhausentgeltgesetz, KHEntgG). Furthermore, the export files generated by the database software are anonymized (e.g., case and patient number) for data protection purposes before transmission. IQVIA hospital data have been utilized for a number of prior epidemiological studies [9,10,11].

### 2.2. Study Population and Outcome

**Figure 1:**
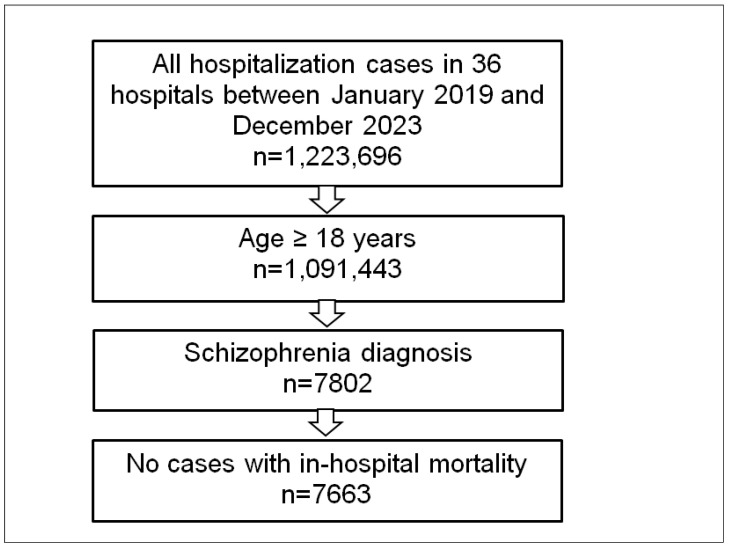
Selection of the study sample.

This study included all hospitalizations of adult patients with a primary or secondary diagnosis of schizophrenia (ICD-10: F20) between January 2019 and December 2023. To ensure comparability and clarity in the analysis, cases with in-hospital mortality were excluded from the study (Figure 1).

In this study, a primary diagnosis of schizophrenia was defined as the main reason for hospitalization, reflecting cases where treatment was primarily focused on managing schizophrenia. In contrast, a secondary diagnosis of schizophrenia refers to cases where schizophrenia was a coexisting condition, identified either at admission or during the hospital stay but not the primary reason for hospitalization. This distinction is clinically important, as it may reflect differences in the severity of schizophrenia or the type of care required. For example, patients hospitalized for primary schizophrenia are likely experiencing acute exacerbations or decompensations requiring specialized care, whereas secondary schizophrenia diagnoses might occur in patients hospitalized for other medical or surgical conditions.

The primary outcome of the study was the prevalence of discharge against medical advice (DAMA). The proportion of hospitalizations with DAMA was calculated for the overall population as well as for subgroups stratified by six age groups (18–30, 31–40, 41–50, 51–60, 61–70, >70 years), sex (women/men), and primary versus secondary diagnosis of schizophrenia. The prevalence of comorbidities documented in at least 5% of the study population was also calculated. These included thyroid gland disorders (ICD-10: E00–E07), diabetes mellitus (ICD-10: E10–E14), lipid metabolism disorders (ICD-10: E78), volume depletion (ICD-10: E86), hypokalemia (E87.6), hypertension (ICD-10: I10), urinary tract infection (ICD-10: R39.0), chronic obstructive pulmonary disease (COPD, ICD-10: J44), respiratory failure (ICD-10: J96), alcohol-related disorders (ICD-10: F10), and cannabis-related disorders (ICD-10: F12).

### 2.3. Statistical Analyses

Baseline characteristics were presented as absolute numbers and proportions (%) for categorical variables and mean value with standard deviation (SD) for age. The normal distribution of the age was assessed using the Kolmogorov–Smirnov test. Both univariable and multivariable logistic regression analyses were conducted to assess the associations between demographic and clinical variables and DAMA. The dependent binary variable in these models was DAMA. Independent variables included age group, sex, primary versus secondary schizophrenia diagnosis, and the identified comorbidities. First univariable logistic regression was conducted. All variables with a *p*-value < 0.10 were finally included in the multivariable regression analysis. A primary diagnosis of schizophrenia was assumed to reflect hospitalization primarily for the management of schizophrenia, whereas secondary diagnoses indicated coexisting schizophrenia. This distinction was considered important in the regression analysis to account for differences in clinical presentation and management priorities. Additionally, the multivariable logistic regression analysis was conducted for patients hospitalized with schizophrenia as primary diagnosis only, adjusted for age, sex, and comorbidities.

The decision to exclude hospital department (psychiatric vs. somatic) from the multivariable regression model was based on the strong correlation between this variable and the primary versus secondary diagnosis of schizophrenia. Specifically, hospitalizations with a primary schizophrenia diagnosis were overwhelmingly managed in psychiatric departments, while those with secondary schizophrenia diagnoses were often treated in somatic departments. Including hospital departments in the model would, therefore, introduce multicollinearity and potentially obscure the true associations between the independent variables and DAMA.

Results from the logistic regression models were presented as crude odds ratios (cOR) in the univariable regression and adjusted odds ratios (aOR) in the multivariable regression, both with 95% confidence intervals. Due to multiple comparisons, statistical significance was defined based on the Bonferroni correction as *p*-values < 0.0029 (calculated as <0.05/17 models).

Additionally, the DAMA rate for patients with schizophrenia as primary diagnosis was descriptively compared between psychiatric departments (6 out of 36 hospitals). Finally, the DAMA rate was shown for patients hospitalized for the first, second, third, fourth, and fifth time in the period 2018–2023.

All analyses were performed using SAS 9.4 (SAS Institute, Cary, NC, USA).

## 3. Results

### 3.1. Baseline Characteristics

This study included a total of 7663 hospitalization cases of 4356 patients with a mean age of 49.5 years (SD: 17.7), with 40.6% being female. The median length of hospital stay was 10 days. Overall, 51.4% of patients were hospitalized with schizophrenia as the primary diagnosis in six psychiatric departments, while 48.6% were hospitalized for other diagnoses but had schizophrenia listed as a secondary diagnosis in 36 psychiatric and somatic departments. The majority (73.9%) of patients had a diagnosis of paranoid schizophrenia (ICD-10: F20.0), followed by unspecified schizophrenia (ICD-10: F20.9, 13.3%), since other types of schizophrenia (F20.1–F20.8) were rare, with <3% each. Among patients with schizophrenia as the primary diagnosis, 98.5% were treated in the psychiatry department. Hypertension (24.9%), diabetes mellitus (14.9%), and thyroid gland disorders (9.6%) were the most common codiagnoses (Table 1). Of 4356 schizophrenia patients with at least one hospitalization in the period 2018–2023, 1420 patients were hospitalized two times, 696 three times, 390 four times, and 801 more than four times.

### 3.2. Prevalence of Discharge Against Medical Advice

**Figure 2:**
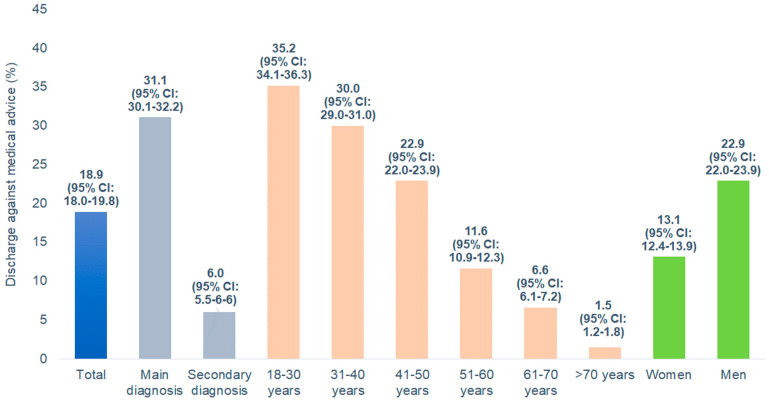
Discharge against medical advice in patients hospitalized for schizophrenia by diagnosis type, age, and sex.

Of the 7663 hospitalization cases included in this study, some 1447 (18.9%; 95% CI: 18.0–19.8) were recorded as DAMA. The DAMA rate was 31.1% (95% CI: 30.1–32.2) in patients with schizophrenia as the primary diagnosis and 6.0% (95% CI: 5.5–6.6) in patients with schizophrenia as a secondary diagnosis. Furthermore, the DAMA rate decreased from 35.2% (95% CI: 34.1–36.3) in the age group 18–39 years to 1.5% (95% CI: 1.2–1.8) in the age group over 70 years and was higher among men (22.9%; 95% CI: 22.0–23.9) than among women (13.1%; 95% CI: 12.4–13.9) (Figure 2). The median length of hospital stay was 8 days for DAMA patients and 10 days for non-DAMA patients. Overall, 47.5% (95%: 46.4–48.6) left the hospital within one week after admission to the hospital (11.3% on the 1st day, 13.0% on the 2nd day, 14.6% between the 8th and 14th day, 10.1% between the 15th and 21st day, 7.9% between the 22nd and 28th day, 13.0% within the 2nd month, and 3.9% within the 3rd month).

**Figure 3:**
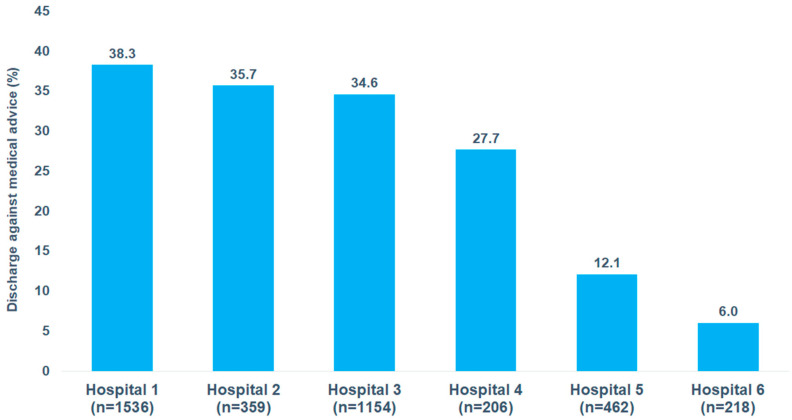
Discharge against medical advice in patients hospitalized for schizophrenia by hospital.

Figure 3 shows the DAMA rate among patients hospitalized with schizophrenia as primary diagnosis in six psychiatric departments. The DAMA rates were strongly different and varied between 6.0% and 38.3%, depending on the hospital.

**Figure 4:**
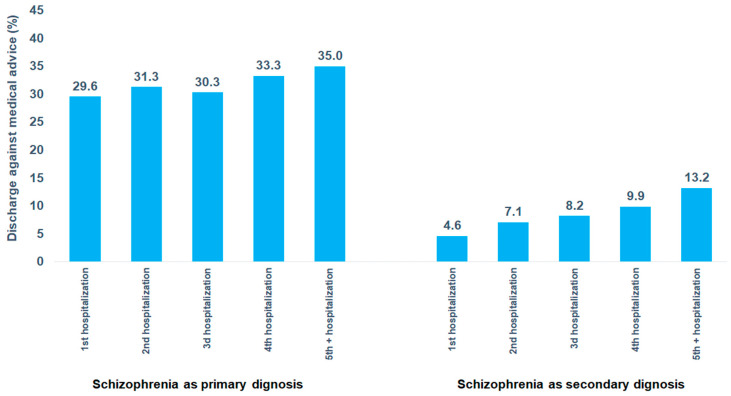
Discharge against medical advice in patients hospitalized for schizophrenia as primary diagnosis by psychiatric department.

Figure 4 shows the DAMA rate among patients hospitalized with schizophrenia as either a primary or secondary diagnosis, depending on the number of hospitalizations from 2018 to 2023. Among patients with schizophrenia as a primary diagnosis, the DAMA rate slightly increased from 29.6% after the first hospitalization to 35.0% after the fifth or more hospitalizations. In contrast, among patients with schizophrenia as a secondary diagnosis, the DAMA rate showed a more pronounced increase, rising from 4.6% after the first hospitalization to 13.2% after the fifth or more hospitalizations.

### 3.3. Factors Associated with Discharge Against Medical Advice

In univariable logistic regression, lower age, male gender, schizophrenia as primary diagnosis, psychiatric hospital department, and alcohol-related and cannabis-related disorders were positively associated with a DAMA risk, since all somatic diagnoses were negatively associated with DAMA risk. In the multivariable regression model, several variables were found to be associated with an increased risk of DAMA. Firstly, a clear association between age and DAMA risk was observed, with younger patients exhibiting a stronger association. Specifically, the adjusted odds ratio (aOR) was 7.44 in the age group 18–30 and 2.73 in the age group 61–70, compared to the group of patients aged over 70 (*p* < 0.0001). In addition, schizophrenia as the primary diagnosis was strongly associated with DAMA risk compared to schizophrenia as a secondary diagnosis (aOR: 3.61; 95% CI: 3.05–4.26, *p* < 0.0001). Furthermore, alcohol-related disorders (aOR: 1.68; 95% CI: 1.38–2.04, *p* < 0.0001) and cannabis-related disorders (aOR: 1.43; 95% CI: 1.18–1.72, *p* < 0.0001) were positively associated with DAMA. Conversely, certain secondary diagnoses, including hypertension (aOR: 0.61; 95% CI: 0.49–0.76, *p* < 0.0010) and respiratory failure, (aOR: 0.32; 95% CI: 0.16–0.65, *p* < 0.0001) were significantly associated with a decreased risk of DAMA (Table 2).

When multivariable regression has been conducted for patients hospitalized with schizophrenia as the primary diagnosis, only age groups 18–30 years (aOR: 4.83; 95% CI: 2.17–10.73) and 31–40 years (aOR: 4.46; 95% CI: 2.01–9.89) were significantly associated with an increased risk of DAMA, since hypertension diagnosis was significantly associated with a decreased risk of DAMA (Table 3).

## 4. Discussion

The present study investigated the prevalence of and factors associated with DAMA among hospitalized patients, revealing significant insights into the characteristics and conditions contributing to DAMA rates. Our study is the first in Germany and probably the first in Europe to report the DAMA rate in schizophrenia patients.

Based on a study population of 7663 hospitalizations of patients diagnosed with schizophrenia, the DAMA rate was found to be 31.1% in patients with schizophrenia as the primary diagnosis and 6.0% in those with schizophrenia as a secondary diagnosis. In addition, factors such as younger age and alcohol- and cannabis-related disorders were found to be associated with an increased risk of DAMA.

The DAMA rate in the present study is higher than in previous studies [5,6,7]. The highest DAMA rate reported yet was in the study by Wung et al. with 18.7% [7]. The results from German hospitals cannot be directly compared with studies from the US, Israel, and Taiwan due to differences in healthcare systems, hospital policies, cultural attitudes toward psychiatric treatment, and legal regulations regarding patient discharge. Variations in insurance coverage, availability of community-based mental health support, and criteria for hospitalization may also influence DAMA rates. Additionally, differences in study methodologies, sample characteristics, and definitions of DAMA further limit direct comparability. While there may not be a single clear explanation for the large difference between the present findings from Germany and findings from the US, there are some possible factors that could contribute to this discrepancy. In many US states, involuntary psychiatric hospitalization laws are relatively strict, and patients can be held longer if they are considered a danger to themselves or others. Moreover, many US hospitals have locked psychiatric wards, making it physically harder to leave. Furthermore, some patients in the US may fear losing health insurance benefits if they leave AMA, making them stay. Finally, US hospitals may use stronger persuasion techniques to prevent DAMA.

Age emerged as a significant variable strongly associated with DAMA, with younger patients displaying a higher likelihood of DAMA than older patients. This age-related pattern may reflect various psychosocial and behavioral factors, including a perception of lower vulnerability to health complications, a desire for autonomy, and a higher likelihood of non-compliance with medical advice. Younger patients may have more severe schizophrenia. Kao and Liu demonstrated that patients with early-onset schizophrenia exhibit significantly higher levels of cognitive impairment and greater impulsivity [12]. In a meta-analysis based on 81 studies, authors reported a positive association between young age and severity of negative symptoms as well as frequency of relapses and poorer social functioning among schizophrenia patients [13]. Regarding the role of age in DAMA risk, our study is in line with the findings of Sclar and Robison from the USA, who found that DAMA patients were significantly younger than non-DAMA patients [6].

The presence of schizophrenia as the primary diagnosis was strongly associated with DAMA. In our study, patients with schizophrenia as their primary diagnosis were primarily treated in psychiatric departments. Treatment for schizophrenia is necessary when patients were in a phase of decompensation or experienced an acute relapse. In this phase, schizophrenia is often accompanied by impaired judgment, paranoia, and a lack of insight into one’s condition, which can lead to non-compliance with medical advice [14,15].

Conversely, patients with schizophrenia as a secondary diagnosis were treated for acute or chronic physical illnesses, including hypertension, lipid metabolism disorders, or infectious diseases. It could be assumed that these patients had compensated schizophrenia without acute symptoms; in addition, their physical condition was weakened due to other illnesses or surgery. Both factors might contribute to patients staying in the clinic to complete their treatment.

Alcohol- and cannabis-related disorders were also found to be variables that were significantly associated with DAMA. Substance use disorders can exacerbate psychiatric symptoms, impair judgment, and lead to erratic behaviors, all of which can increase the likelihood of DAMA. Patients with substance use disorders might experience withdrawal symptoms or cravings that motivate them to leave the hospital to access substances. Interestingly, substance use disorders are common among patients with schizophrenia and may increase the risk of acute relapse [16,17]. Historically, the literature has consistently demonstrated that substance use disorders are strongly linked to an increased risk of leaving the hospital against medical advice, extending beyond patients with psychiatric disorders [18]. Based on the positive association between alcohol and cannabis use disorders and DAMA observed in the present study, we can confirm this relationship among schizophrenia patients in German hospitals.

Another important finding in the present study is the increase in the DAMA rate with the number of hospitalizations. This could indicate growing dissatisfaction with hospitalization experiences on the one side and worsening psychiatric symptoms on the other side. Previous research has already shown that a history of DAMA may serve as a strong predictor for future occurrences, not necessary in schizophrenia patients only [19,20,21].

Interestingly, the DAMA rate among patients with a primary diagnosis of schizophrenia varied by hospital, ranging from 6.0% to 38.3%, highlighting the hospital’s role in influencing the risk of DAMA. This variation in DAMA rates across hospitals suggests that institutional factors play a crucial role in patient retention and adherence to treatment. This could be due to differences in hospital policies, quality of care, and staff–patient interactions. Some hospitals may have stronger patient engagement strategies or more effective crisis intervention teams, reducing the likelihood of DAMA.

The high rate of DAMA among schizophrenia patients can be attributed to a complex interplay of biological factors, including impaired insight, cognitive deficits, neurotransmitter dysregulation, and emotional dysregulation. For example, one of the hallmark symptoms of schizophrenia is an anosognosia, meaning an impaired insight into one’s condition. Anosognosia is thought to be linked to dysfunction in the prefrontal cortex, which is involved in self-awareness, judgment, and executive functioning. Patients may also experience difficulties in perceiving reality, leading them to reject hospitalization or treatment [22].

The findings of this study have important implications for healthcare practice. There is a clear need for targeted interventions to reduce DAMA rates among high-risk groups, particularly younger patients and individuals with substance use disorders. Schizophrenia patients with primary diagnoses may require more intensive monitoring and engagement strategies during hospitalization to mitigate the risk of DAMA. Clinicians should consider the severity of symptoms, patient insight, and engagement levels when planning treatment and discharge. Hospitals and psychiatric departments should assess their internal practices and environment to identify factors contributing to higher DAMA rates. Improving communication between staff and patients, providing patient-centered care, and ensuring that patients’ concerns are addressed in a supportive and respectful manner may reduce DAMA.

Further research and qualitative studies are needed to explore the underlying reasons as to why patients choose DAMA, particularly among different age groups. Qualitative studies involving patient interviews could provide deeper insights into the personal, social, and economic factors influencing their decisions. In addition, investigating the long-term outcomes of patients who discharge themselves against medical advice could provide valuable insights into the broader impacts of DAMA on health and healthcare systems.

### Limitations

The present study is subject to several limitations inherent in the IQVIA hospital database. Firstly, the diagnoses and codiagnoses relied exclusively on the ICD-10 classification system, and no information was available regarding the clinical severity of schizophrenia. Although we assumed that a primary diagnosis of schizophrenia likely reflects a state of decompensation requiring hospitalization, this assumption cannot be validated with the available data. The absence of specific indicators for disease severity, such as psychometric scales or clinician assessments, limits the study’s ability to explore how varying levels of illness severity might influence discharge against medical advice (DAMA).

Secondly, the data set does not include information on whether hospitalizations were voluntary or involuntary. Given that involuntary treatment is a critical issue in psychiatry and may influence patient behaviors, such as DAMA, the absence of this variable limits the scope of the study. For example, involuntary hospitalization may reduce the likelihood of DAMA in some cases but could also increase resistance to care, leading to a higher risk of DAMA in others. Future studies should aim to include data on the legal status of hospitalization to better explore its relationship with DAMA.

Thirdly, this study does not examine whether the hospitalization was due to the first-episode schizophrenia (FES) or subsequent episodes, as the database only contained data from 2018 to 2023 and no individual patient’s histories prior to 2018.

Fourthly, addiction disorders may be undercoded in the database, as patients with substance use issues often do not disclose or acknowledge their addictions. This limitation could lead to an underestimation of the prevalence of addiction-related comorbidities in the study population. Since addiction disorders, such as alcohol or cannabis use disorders, are known risk factors for DAMA, undercoding could potentially bias the observed associations. Future research could mitigate this limitation by incorporating data from addiction treatment services or by using screening tools to capture substance use that is not formally documented.

Fifthly, the absence of socioeconomic variables, such as family situation, employment status, or income, is another key limitation. Socioeconomic factors are known to influence health outcomes and healthcare-seeking behaviors, including DAMA, but these variables could not be examined in the present study. Including such data in future analyses would provide deeper insights into the social determinants of DAMA.

Sixthly, the lack of data on medication use limits the ability to analyze the potential influence of pharmacological treatments on DAMA. For example, certain antipsychotics or adjunctive medications may reduce the likelihood of DAMA by improving symptoms or reducing side effects, but this hypothesis could not be tested in the present study. Future research should integrate medication data to explore the role of treatment strategies in preventing DAMA.

Seventhly, as the data set only contains information from hospital settings, the findings are not generalizable to the outpatient population. This limitation restricts the study’s applicability to settings, such as outpatient psychiatric clinics or community mental health services, where DAMA may be driven by different factors.

Lastly, the study could not investigate the consequences of DAMA, as the database only includes information from the duration of the hospital stay and does not capture post-discharge outcomes. This limitation prevents an assessment of the potential impacts of DAMA, such as rehospitalization, worsening of symptoms, or mortality. Future studies should include follow-up data to evaluate the long-term outcomes of DAMA and its broader.

## 5. Conclusions

This study highlights the high prevalence of DAMA among hospitalized schizophrenia patients and identifies that younger age, schizophrenia as primary diagnosis during the hospitalization, alcohol-related disorders, and cannabis-related disorders are significantly associated with DAMA risk.

DAMA among schizophrenia patients is a serious clinical challenge for German healthcare system including both inpatient and outpatient care. Addressing the high DAMA rates requires multifaceted approaches, including tailored interventions and enhanced support services. Further studies are needed to explore the reasons for DAMA.

## Figures and Tables

**Table 1:** Baseline characteristics of the study sample.

Variable	Hospitalizations (N = 7663)
Mean age (standard deviation)	49.5 (17.7)
18–30	1335 (17.4)
31–40	1450 (18.9)
41–50	1264 (16.5)
51–60	1368 (17.9)
61–70	1177 (15.4)
>70	1069 (14.0)
Female	3110 (40.6)
Male	4553 (59.4)
Schizophrenia as primary diagnosis	3935 (51.4)
Schizophrenia as secondary diagnosis	3728 (48.6)
Schizophrenia ICD-10 codes	
Paranoid schizophrenia (F20.0)	5666 (73.9)
Schizophrenia, unspecified (F20.9)	1021 (13.3)
Other schizophrenia diagnoses (F20.1–F20.6)	976 (12.7)
Hospital department	
Psychiatry	4272 (55.8)
Somatic departments	3391 (44.2)
Length of hospital stay in days (median, IQR)	10 (25)
Codiagnoses	
Thyroid gland disorders	735 (9.6)
Diabetes mellitus	1144 (14.9)
Lipid metabolism disorders	505 (6.6)
Volume depletion	460 (6.0)
Hypokalemia	665 (8.7)
Hypertension	1910 (24.9)
Urinary tract infection	506 (6.6)
COPD	523 (6.8)
Respiratory failure	517 (6.8)
Alcohol-related disorders	683 (8.9)
Cannabis-related disorders	652 (8.5)

Data are absolute numbers (percentages) unless otherwise specified.

**Table 2:** Association of demographic and clinical variables with discharge against medical advice in patients hospitalized with schizophrenia (multivariable logistic regression).

	Univariable Regression		Multivariable Regression	
Variable	cOR (95% CI)	*p* Value	aOR (95% CI) *	*p* Value
Age group				
Age 18–30 years	35.75 (21.55–59.32)	<0.0001	7.44 (4.35–12.73)	<0.0001
Age 31–40 years	28.20 (17.00–46.79)	<0.0001	6.63 (3.89–11.29)	<0.0001
Age 41–50 years	19.50 (11.70–32.51)	<0.0001	5.59 (3.28–9.54)	<0.0001
Age 51–60 years	8.65 (5.14–14.56)	<0.0001	3.41 (1.99–5.85)	<0.0001
Age 61–70 years	4.67 (2.71–8.05)	<0.0001	2.73 (1.57–4.77)	<0.0005
Age > 70 years	Reference		Reference	
Sex				
Female	Reference		Reference	
Male	1.97 (1.74–2.24)	<0.0001	1.07 (0.93–1.24)	0.3248
Schizophrenia diagnosis type				
Schizophrenia as primary diagnosis	7.01 (6.03–8.15)	<0.0001	3.61 (3.05–4.26)	<0.0001
Schizophrenia as secondary diagnosis	Reference		Reference	
Hospital department				
Psychiatric hospital department **	16.86 (13.56–20.97)	<0.0001	-	
Somatic hospital department **	Reference		-	
Further diagnoses of schizophrenia patients				
Thyroid gland disorders	0.38 (0.29–0.49)	<0.0001	0.75 (0.56–1.01)	0.0518
Diabetes mellitus	0.31 (0.25–0.39)	<0.0001	0.98 (0.76–1.27)	0.8855
Lipid metabolism disorders	0.25 (0.17–0.36)	<0.0001	0.53 (035–0.79)	0.0020
Volume depletion	0.22 (0.15–0.34)	<0.0001	1.05 (0.67–1.66)	0.823
Hypokalemia	0.48 (0.37–0.62)	<0.0001	1.26 (0.94–1.69)	0.1185
Hypertension	0.24 (0.19–0.28)	<0.0001	0.61 (0.49–0.76)	<0.0001
Urinary tract infection	0.13 (0.08–0.22)	<0.0001	0.44 (0.26–0.74)	0.0019
COPD	0.20 (0.14–0.30)	<0.0001	0.74 (0.47–1.14)	0.1653
Respiratory failure	0.07 (0.04–0.14)	<0.0001	0.32 (0.16–0.65)	0.0001
Alcohol-related disorders	2.25 (1.89–2.67)	<0.0001	1.68 (1.38–2.04)	<0.0001
Cannabis-related disorders	3.48 (2.94–4.11)	<0.0001	1.43 (1.18–1.72)	<0.0001

* Multivariable logistic regression adjusted for age, sex, diagnosis type, and codiagnoses; cOR = crude odds ratio; aOR = adjusted odds ratio. ** Hospital department (psychiatric/somatic) were not included in the multivariable regression model, as this variable has a strong correlation with primary/secondary diagnosis of schizophrenia.

**Table 3:** Association of demographic and clinical variables with discharge against medical advice in patients hospitalized with schizophrenia as primary diagnosis (multivariable logistic regression).

Variable	OR (95% CI) *	*p* Value
Age group		
Age 18–30 years	4.83 (2.17–10.73)	0.0001
Age 31–40 years	4.46 (2.01–9.89)	0.0002
Age 41–50 years	3.79 (1.70–8.41)	0.0011
Age 51–60 years	2.75 (1.23–6.15)	0.0138
Age 61–70 years	2.53 (1.11–5.80)	0.0279
Age > 70 years	Reference	
Sex		
Female	Reference	
Male	1.06 (0.90–1.25)	0.4739
Codiagnoses		
Thyroid gland disorders	0.68 (0.48–0.95)	0.0239
Diabetes mellitus	0.95 (0.68–1.31)	0.7367
Lipid metabolism disorders	0.60 (0.39–0.94)	0.0243
Volume depletion	1.06 (0.52–2.16)	0.8828
Hypokalemia	1.42 (0.99–2.03)	0.0537
Hypertension	0.58 (0.45–0.75)	<0.0001
Urinary tract infection	0.41 (0.21–0.81)	0.0099
COPD	0.44 (0.21–0.90)	0.0236
Respiratory failure	0.38 (0.05–3.00)	0.3585
Alcohol-related disorders	1.25 (0.99–1.58)	0.0640
Cannabis-related disorders	1.29 (1.05–1.58)	0.0159

* Multivariable logistic regression adjusted for age, sex, and codiagnoses.

## Data Availability

The data and code used for this study are available from the corresponding author upon reasonable request. The data belong to the company IQVIA, and the company does not share the data, as these data cost money; we cannot share data we obtained for free due to our employment.
